# Supplementary material for: No evidence for a mixing benefit—A registered report of voluntary dialect switching
Source: PLoS One. 2023 May 4;18(5):e0282086. doi: 10.1371/journal.pone.0282086 (PMC10159105; doi:10.1371/journal.pone.0282086)
Supplement: S1 Table — (PDF) [file pone.0282086.s001.pdf]

Standard English	Dundonian
Armpit	Oxter
Boy	Laddie
Cast	Stookie
Children	Bairns
Chimney	Lum
Ears	Lugs
Earwig	Forkytaily
Face	Pus
Girl	Lassie
Hill	Brae
Kerb	Cribby
Lake	Loch
Notebook	Jotter
Onion	Ingin
Potato	Tattie
Sandwich	Piece
Slippers	Baffies
Spot	Plook
Stream	Burn
Turnips	Neeps
