# Supplementary material for: No evidence for a mixing benefit—A registered report of voluntary dialect switching
Source: PLoS One. 2023 May 4;18(5):e0282086. doi: 10.1371/journal.pone.0282086 (PMC10159105; doi:10.1371/journal.pone.0282086)
Supplement: Attachment — Submitted filename: Revision - PONE-D-22-14768R1 - Response to Reviewers.docx [file pone.0282086.s003.docx]

Dear Editor,

We are sending you the revised version of our manuscript named “No evidence for a mixing benefit - A Registered Report of voluntary dialect switching”. We are grateful for the comments by the reviewers and incorporated them where possible.

A detailed point-to-point response to the questions of the reviewers is provided below. Where appropriate a reference to the respective changes in the manuscript is given and the major changes are highlighted in the manuscript.

Reviewer 1

**1.** P6: revised “have shown” should be “have been shown”

**Reply:** We have made the suggested amendment.

**2.** P11: After reading reviewer 2’s comment and then looking at the revision, I am still confused about the single language blocks. From reviewer 2’s comment, it sounds like the standard design is 2 single language blocks (one in each language), followed by the switching blocks, followed by 2 single language blocks again (one in each language). If that is the standard design in previous research, the authors should clarify why they chose to present only one language block before and only one language block after the switching.

**Reply:** There is no standard in the language switching literature regarding block type order (for a discussion, see Declerck, 2020). Even when solely focusing on voluntary language switching, many different approaches have been used: Some voluntary language switching studies that relied on single and mixed language blocks started with a single language block of each language, followed by the mixed language block(s), and then again a single language block of each language (e.g., Jevtović et al., 2020). Another voluntary language switching study completely counterbalanced all possible block type orders across participants (Gollan & Ferreira, 2009), while another had both single language blocks consistently prior to the mixed language block (Grunden et al., 2020). One study even conducted the single language and mixed language blocks in different sessions one week apart, with the single language blocks performed in the first session and the mixed language blocks in the second session (Gross et al., 2015). While these bilingual voluntary language switching studies all had a different approach regarding the block type order, they all showed a mixing benefit. So, it is unlikely that the mixing benefit observed in voluntary language switching studies is due to a specific block type order.

**3.** P21: My initial comment about this sentence was that it was difficult to understand, and I don’t think that the revision made it easier to understand. I would advise breaking the ideas up into smaller sentences. It may also be helpful to specifically name the “underlying mechanism that turns a mixing cost into a mixing benefit.” I think you might mean a reduction in proactive control. If so, I would advise something like this: “Some researchers have proposed that the underlying mechanism that explains the difference between mixing costs during cued bilingual language switching and mixing benefits during voluntary bilingual language switching is a difference in proactive control. In other words, cued bilingual language switching results in mixing costs because it requires more proactive control (relative to single-language use), but voluntary bilingual language switching results in mixing benefits because it requires less proactive control (relative to single-language use). For bidialectals, the amount of proactive control needed to switch languages, relative to using a single language, may be different than for bilinguals, leading to a reduction in both mixing costs and mixing benefits.”

**Reply:** Unfortunately, this is not exactly what we meant. We have changed the text to further elaborate and clarify the idea we suggested in the previous version (pages 20-21). In short: We assume that more control processes are required in a bilingual involuntary setting than in a voluntary bilingual setting, leading to different mixing effect patterns (i.e., mixing benefit and mixing costs, respectively). A similar, but less pronounced, difference could probably be observed with bidialectals, leading to a mixing cost pattern in a bidialectal involuntary setting and no mixing effect in a bidialectal voluntary setting.

Because it is unclear what the main underlying mechanism is of mixing costs (e.g., de Bruin et al., 2018; Ma et al., 2016), and because our results do not allow for any speculation regarding this underlying mechanism, we did not provide any specifics about which underlying mechanism this is attributed to.

Reviewer 2

**1.** Thank you for making these revisions and for addressing my previous comments! I'm happy for this manuscript to be accepted in its current form.

**Reply:** Thank you for the time and effort you put into your review, which has substantially elevated our manuscript!

We would like to thank you and the reviewers for these helpful comments. We hope you find our revision and response to the reviewers’ comments satisfactory.

Sincerely,

Mathieu Declerck and Neil W. Kirk
